# Clinical relevance of the compartments and lymphocyte subsets in the human spleen

**DOI:** 10.1007/s00441-025-04001-0

**Published:** 2025-08-29

**Authors:** Reinhard Pabst

**Affiliations:** Institute of Immunomorphology, Centre of Anatomy, Medical School Hannover, Carl-Neuberg-Straße 1, 30625 Hannover, Germany

**Keywords:** Spleen, Compartments, Splenectomy, Postsplenectomy, Infection, Man

## Abstract

The compartments and lymphocyte subsets of the human spleen differ from the spleen of rodents. The red pulp removes old red cells or malformed erythrocytes found in spherocytosis. The high blood flow is also necessary to filter bacteria like pneumococci from the blood. Without a spleen or after splenectomy, there is the risk of a fatal postsplenectomy sepsis. Therefore, these patients have to be vaccinated. Splenic particles can regenerate. The spleen is very important in lymphocyte recirculation and lymphocyte production. The marginal zone B lymphocytes are unique and important for B memory in man.

Dedicated to Prof. Jürgen Westermann for decades of cooperation in research and teaching of functional clinically relevant anatomy.

## Introduction: normal human spleen

More than 400 years ago, Ballonius asked the rhetorical question, is the spleen necessary for life (Pabst 1981). Meanwhile, there is a general agreement (Sherman 1980) on the clinical relevance of the spleen in humans.

**Fig. 1:**
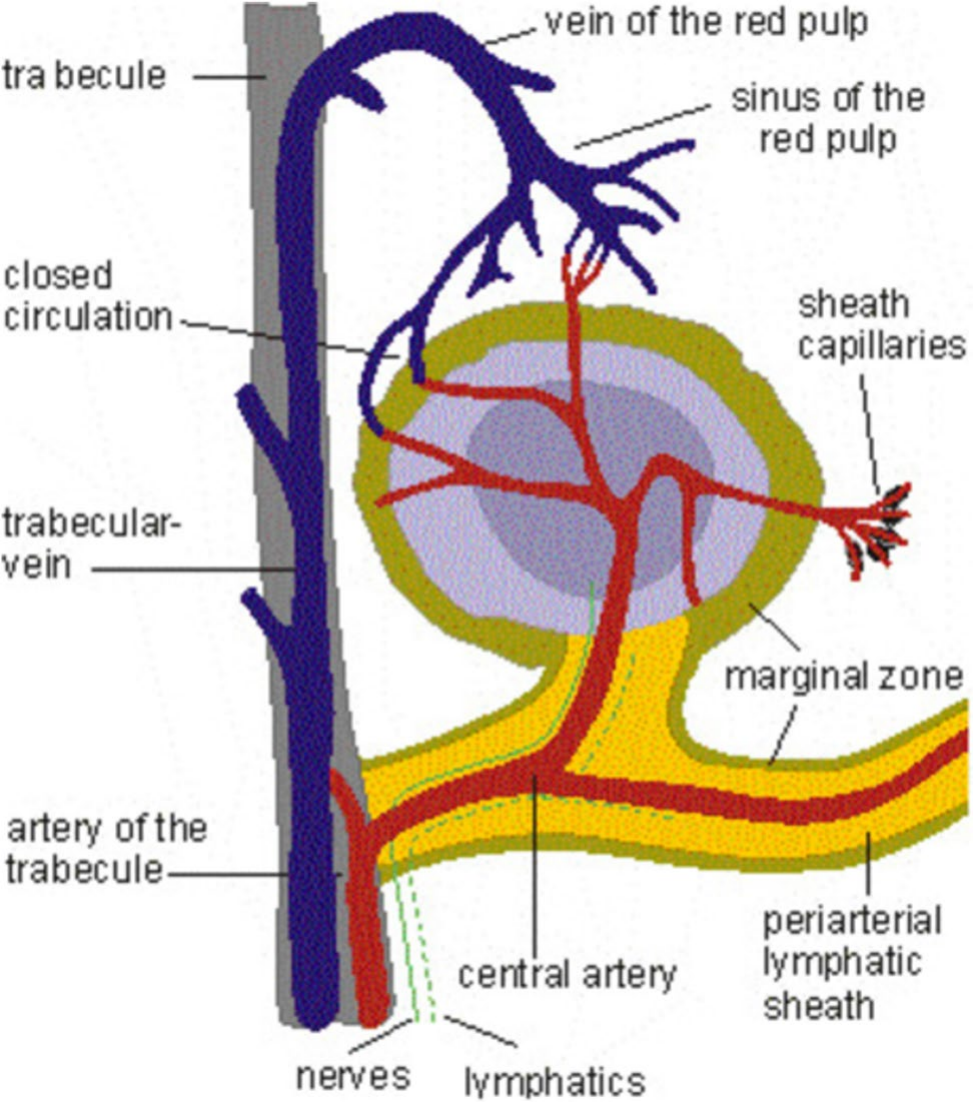
Schematic drawing of the white pulp of the human spleen. The T cell zone is the periarteriolar area (PALS). The germinal center is surrounded by the marginal zone of the B cell area. The capillary sheaths are strongly positive for CD 169. The arrows indicate the blood flow

The spleen is the largest secondary lymphoid organ; although only 0.3% of body weight, the spleen receives 3.5% of the cardiac output. The blood supply is enormous not for oxygen consumption, but for the splenic function to clear the blood from particular antigens and microorganisms, as well as clearing aged red cells. The spleen has no afferent lymphatics, but there are efferent lymphatics starting in the capsule and running along the trabecular arteries to the hilum, where they drain into the splenic hilar lymph nodes (van Krieken et al. 1985) (Fig. 1). The weight of the spleen increases up to an age of 5 years. Then, it is constant, and there is no decrease in weight with age (van Krieken et al. 1985). Steiniger et al. (2007) have described the fetal and early postnatal development of the human spleen.

The innervation of the spleen is mainly by the sympathetic but also by parasympathetic nerves. The nerves run around the splenic artery. They seem to have all afferent functions (Fig. 1).

The spleen is found in the left upper abdominal cavity and can easily be examined by ultrasound. The normal size (4 × 7 × 11 cm) is of relevance, e.g., for the decision on contact sports after mononucleosis. There is an obvious correlation of body height and splenic size as documented in a cohort of 1230 volunteers (Chow et al. 2016).

The spleen plays an important role in lymphocyte migration because more lymphocytes leave the blood in the spleen per day than in all lymph nodes (Pabst 1998). The T lymphocyte subset distribution in the human spleen differs from the peripheral blood, and it has fewer CD4^+^ T cells, resulting in an inverse CD4/CD8 ratio (Langeveld et al. 2006). There are proportionally more B cells in the spleen. Montorsi et al. (2022) have reviewed the localization of B cells in human lymphoid organs recently. I will focus on the human spleen and recommend differences between the mouse and human spleen to the review of Mebius and Kraal (2005).

### Compartments of the spleen

The majority of the splenic parenchyma (_~_75%) is the red pulp. The lymphoid white pulp consists of the perifollicular lymphatic sheath (PALS) with a predominance of T cells and the lymphoid nodules with mainly B lymphocytes. The third splenic compartment is the marginal zone (Fig. 1).

#### Red pulp

**Fig. 2:**
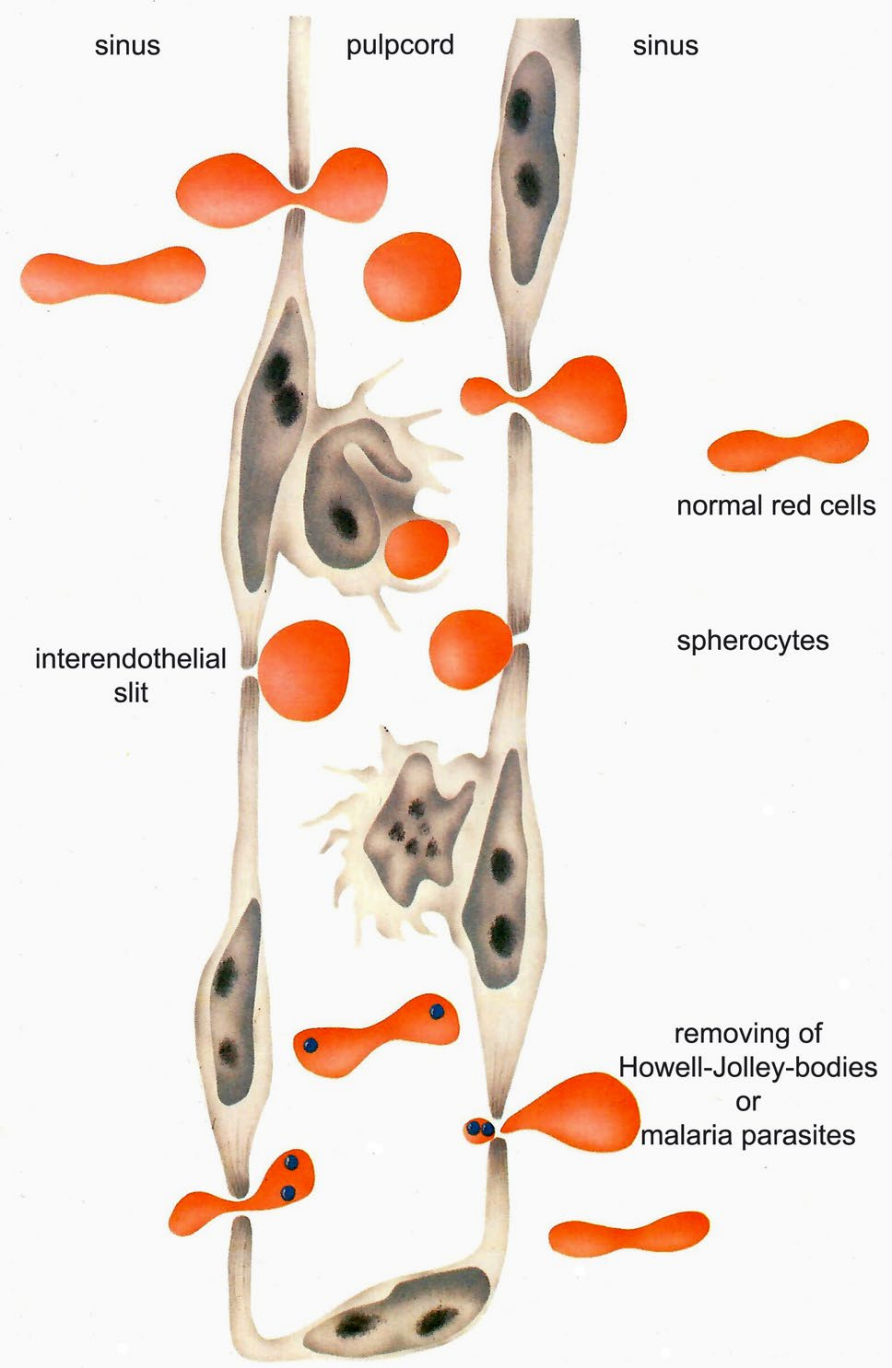
Schematic drawing of the red pulp. The endothelium is supported by reticular fibers (ring fibers). Leukocytes, thrombocytes, and red cells enter the sinus of the red zone through slits. These are removed by remnants of nuclear substance of red cells (Howell Jolley bodies) or by malaria-infected red cells

The main function of the red pulp is to remove particles from the erythrocytes, e.g., the Howell-Jolly bodies, which are remnants of the nucleus of the red cell precursors. The red cell membrane contains tiny vacuoles (pitted red cells), which can be identified by interference contrast microscopy. When a patient is infected by malaria, the red cells partly contain these parasites. All these inclusions can be removed by an intact red pulp (Fig. 2).

The mechanisms of filtering *Plasmodium falciparum*-infected red cells have been studied by the extracorporeal perfusion of human spleens. Contrast-enhanced ultrasound examinations are another modern technique to evaluate splenic perfusion (Omar and Freemann 2016).

There has been a long debate whether the red pulp has an open or closed or both type of microcirculation. In a study of the human splenic red pulp, it is clearly documented that the human spleen has an entire open circulation (Steiniger et al. 2011). In the red pulp in man, there is also a pooling of platelets and phagocytosis of defective (e.g., spherocytes) or old red cells (Fig. 2).

Asplenia is the absence of the spleen (rarely congenital mostly after a splenectomy). Hyposplenism is a defect in splenic function, e.g., due to hematological diseases, autoimmune disorders, or gastrointestinal disorders (Lenti et al. 2022). The opposite is hypersplenism. This can be due to an enlarged spleen in many diseases, e.g., lymphoma or congestion in portal hypertension. This can be treated by partial spleen embolization (Meine et al. 2021).

The red pulp is relevant for iron recycling: Old erythrocytes are removed by macrophages. There is no storage function for red cells in the human spleen. The human erythrocyte has an average life span of about 120 days. In other species, such as dogs and seals, large amounts of red cells are stored in the spleen at rest. The contraction of muscular trabeculae and the capsule releases these red cells into the circulation, resulting in a dramatic increase in the capacity to transport oxygen.

The red pulp has an important role in monitoring physiological levels of platelets. There are about 1/3 of all platelets at a given time in the red pulp. Therefore, hypersplenism can lead to thrombocytopenia in the blood (Brendolan et al. 2007).

The interendothelial slits in the red pulp of the spleen determine the size and shape of erythrocytes. By computational stimulation, Pivkin et al. (2016) described the basis for the removal of red cells infected with *Plasmodium falciparum*. Mannose-binding lectins, including the mannose receptor CD26, can be used to test patients for potential functional hypersplenism.

#### White pulp

There are at least three phenotypically and morphologically distinguishable types of branched stromal cells in the white pulp of the human spleen (Steiniger et al. 2014).

Another spleen-specific structure in man is capillary sheaths consisting of CD22^+^ stromal cells, CD68^+^, CD65-macrophages, and recirculating B lymphocytes as documented by combining immunohistology with 3D virtual models (Steiniger et al. 2018).

All secondary lymphoid organs such as lymph nodes, tonsils, and Peyer’s patch have primary lymphoid follicles, which form secondary follicles after antigen contact. In the human spleen, the secondary follicles are not asymmetric polarized and do not contain a dark and light zone as in rodents. The typical stromal cells of the B cell follicles are the follicular dendritic cells (FDC). These have been localized in the human spleen by Steiniger et al. (2011).

#### Marginal zone

**Fig. 3:**
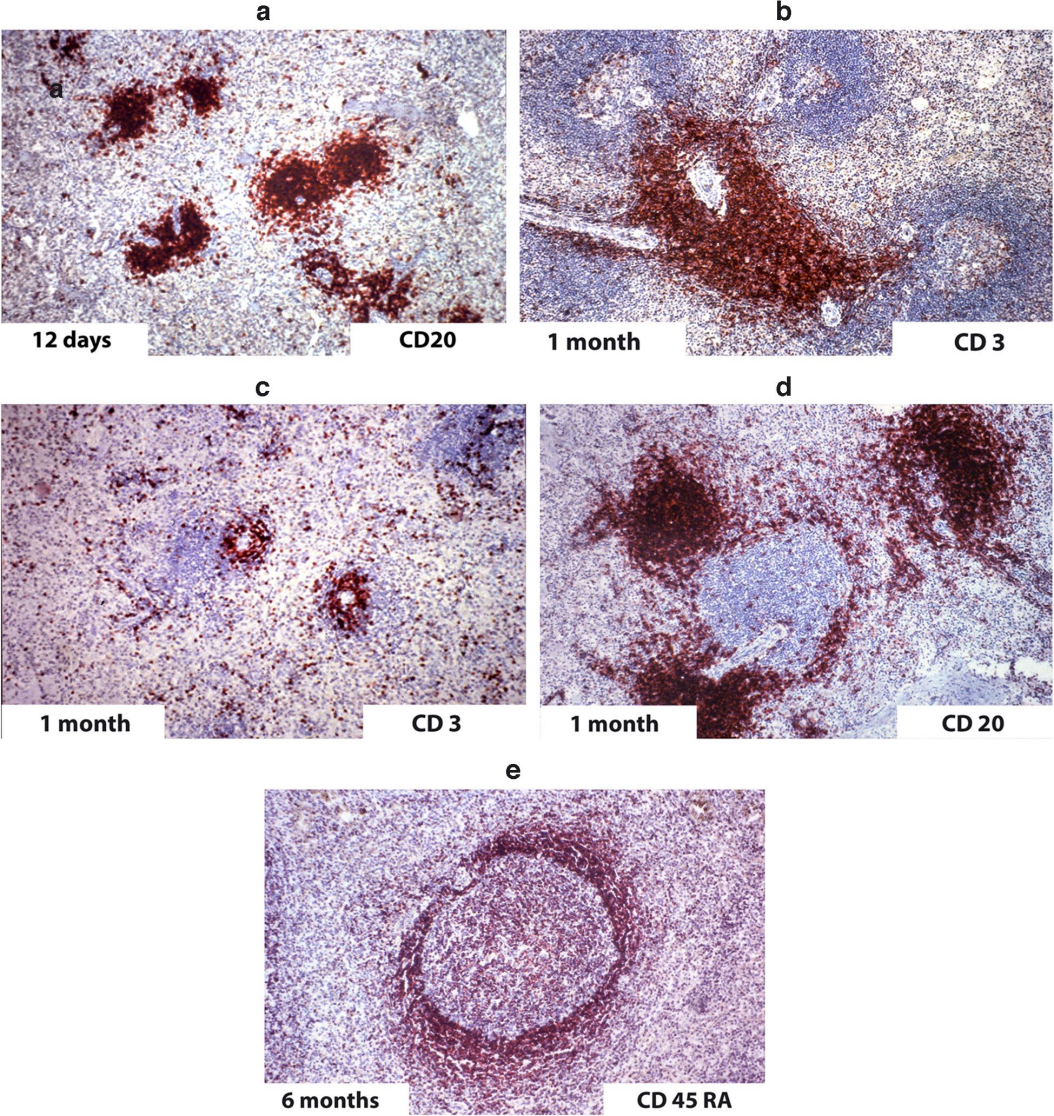
Immunohistology of spleens of children of different ages. **a** 12 days; **b, c, d** 1 month; **e** 6 months of age (magnification 80 ×)

The marginal zone of the human spleen is immature in early infancy. Timens et al. (1989) studied 32 infant spleens (< 2 years) and six spleens of children. The marginal zone showed some delay in its development in contrast to the other compartments, which reach the structure of the adult spleen at an age of 5 months. The marginal zone of infants lacks the B cells (CD24^+^) and high expression of IgM and IgG on B cells in this area. The marginal zone was often missing in the spleens of children who died of the sudden infant death syndrome (Kruschinski et al. 2004) (Fig. 3). The immaturity of the marginal zone seems to be the reason for insufficient infant immune response (Timens et al. 1989). Using the marker of CD27, it was documented that naive B cell populates the infant marginal zone cells (Zandvoort et al. 2001).

#### Marginal zone B cell

The cell biology of the marginal zone of the spleen is very complex (Kraal and Mebius 2006). The B cells in the marginal zone are so unique that they will be discussed in this separate paragraph. The differences between mouse and human marginal zone B cells have been discussed. In man, these cells are typically IgM^hi^, Ig D^lo^, CD27^hi^, and CD23^−^. Human marginal zone B cells express high levels of CD1c in contrast to the mouse; that B cells recirculate and are said to be the first line of defense against blood-born pathogens (Zandvoort and Timens, 2002), particularly encapsulated bacteria (Pillai et al. 2005). Already in 1995, the group of Joe Spencer analyzed the mutations in Ig heavy chain region genes and concluded these B cells are a reservoir of memory B cells (Dunn-Walters et al. 1995). It is of interest that sinus lining cells in the human spleen expressed MAdCAM-1 (van Krieken et al. 1983; Steiniger et al. 2006). This is an adhesion molecule with a critical role in directing lymphocytes to the mucosal-associated lymphoid tissue including mesenteric lymph nodes. The splenic marginal zone develops in the first years of life. For details, see Fig. 3 (Kruschinski et al. 2004; Kibler et al. 2023). This might explain the high rate of pneumococcal sepsis in small children. The splenic marginal zone in man is an archive of memory B cells [28]. They develop from early T2 progenitors (Tull et al. 2021). Siu et al. (2022) described two subsets of marginal zone B cells. They differ in transcriptional profiles; immunoglobulin diversity only marginal zone B cells type 1 are replaced in patients with systemic lupus erythematosus. The clinical importance of marginal zone B cells, in man, is shown by malignant tumors of the mucosal-associated lymphoid tissue (MALT), which are classified as marginal zone B cells because of a complete phenotype morphology and their reactivity with the monoclonal antibody CD12 (Spencer et al. 1998). On the other hand, human spleens from patients with Morbus Hodgkin’s disease without involvement of the spleen and controls were similar (Timens and Poppema 1985).

The group of J. P. Weill et al. (2009) has used human material and studied antivaccine virus antibodies using the knowledge that smallpox vaccination started in the early 1970s and has been eradicated worldwide since 1977. The antivaccine specific IgG^+^ cells comprised a total number of 0.07% of circulating IgG^+^ cells in blood but 0.24% (that is 10–20 million cells) in the spleen of splenectomized patients (7 to 50 years previously) (Chappert et al 2022). These cells were in an extremely low number but not completely absent (Mamani et al. 2008). In a more recent study, the same group characterized these marginal zone B cells as enriched CD21^high^ CD20^high^ Ig cells (Chappert et al. 2022). The situation seems to be different to B cells memory to coronavirus. It is well known that memory of the B cell system relies on a long-lived plasma cell residing in the bone marrow and memory B cells in the spleen (Manz et al. 1998).

Single cell–based gene expression of marginal zone B lymphocytes (CD21^high^, IgM^high^) among healthy organ donors showed that a major splenic marginal zone B cell was absent at birth but also decreased with age. These cells lacked CD27 expression but carried a weak to intermediate memory B cell signet (Kibler et al. 2022). Recently, Tangye et al. (2023) summarized inborn errors of human B cell development within the spleen and the compartments; no differences have been found.

Memory B cells are generated in secondary lymphoid organs throughout life. The splenic marginal zone is the primary site, where memory B cells are generated from a stochastic exchange within the archived memory pool. The clonality increases with age but peaks at midlife. This happens through transient CD2, high expression upon NOTCH2 stimulation. This is studied in human splenic tissue from leftover material of organ donors of a broad range of ages (Kibler et al. 2021).

## Lymphocyte recirculation

The classical route of lymphocyte recirculation is known for lymphoid organs with high endothelial venules as is the T cell area in lymph nodes, tonsils, and Peyer’s patches. Lymphocytes adhere to the endothelium of these special venules and enter the parenchyma. They leave these lymphoid organs via the efferent lymphatics, which finally end in the thoracic duct. In quantitative aspects, many more lymphocytes leave the blood in the spleen and return to the blood vein after about 30 min (Pabst and Westermann 1991). In the spleen, there are no HEV. Lymphocytes leave the blood in the spleen via the central arteries (Golub et al. 2018). The exit from the spleen was not known for long until the marginal time was studied in detail by Martinez-Pomares et al. (2005).

In rodents, there is a marginal sinus around the marginal zone. In man, however, the marginal zone is only defined by an accumulation of mature B cells. Steiniger et al. (2001) documented the presence of mannose receptors in the cells of sinuses of the red pulp. In cysteine-rich domains, surfaced glycans bind the mannose receptor in their sinus-living cells, thus splenic parenchyma. Unfortunately, the authors do not mention the age of the spleen, which is left over from donors for organ transplantation. So far, marginal zone bridging channels have not been described for the human spleen.

## Dendritic cells and macrophages cells in the human spleen

Dendritic cells (DC) collect, process, and present antigens to T cells by regulatory signals to initiate either immune response or tolerance. There are many DC subtypes. Shortman and Liu (2002) reviewed the differences between mouse and human DC. For human splenic DC, they mention a heterogeneity of expression of CD4, CD11b, and CD11c. DC subsets from blood and spleen were similar and resulted in three conventional DC subsets expressing CD11b/c, CD141, and CD16 and the plasmacytoid DC characterized by CD304 expression (Mittag et al. 2011). The generation of human splenic dendritic cells was stimulated by myofibroblasts in vitro studies (Briard et al. 2005).

The distribution of DC in the human spleen was documented by Mcllroy et al. (2001). The marker DEC is an endocytosis receptor critical for the presentation of antigen. This DEC receptor is only found on dendritic cells in the white pulp. In the red pulp, CD68^+^ macrophages were identified. At the border between the red and white pulp, cells expressing the marker DCSIGN (CD 209) were seen (Pack et al. 2008).

In the spleen of organ donors, the localization of DC subsets was studied: HLA.DR^+^ and CD3-14–16-1a-DC were identified. Eighty-one percent of these cells expressed high CD11c. They were found at three locations: the periarterial T cell zones, B cell zones, and marginal zone. Here, they formed a ring of cells surrounding the white pulp, just inside the ring of CD14^+^ macrophages. In some patients, the expression of the activation marker suggests in vivo activation (Mcllroy et al. 2001). The delay in the postnatal development of dendritic cells in the human spleen can be one reason for a delayed vaccine response in early life (Siegrist 2007). In the human spleen, macrophages are found in the perifollicular zone surrounding the endings of arterioles (Mebius and Kraal 2005; Steiniger et al. 2001).

## Natural killer cells

The lymphoid lymphocyte subsets such as T and B lymphocytes are found in an organ-specific localization in lymphoid and non-lymphoid organs. NK cells are morphologically quite similar to lymphocytes; their organ distribution is, however, very different. It has been estimated that about 15% of nucleated cells in the human spleen are NK cells (Westermann and Pabst 1992). In a review, Carrega and Feruazzo (2012) listed many aspects of NK cells in man, which were not known before. Often, subsets of NK cells are not distinguished and differentiated from innate lymphoid cells. There are many aspects of NK cell maturation in the different compartments of the human spleen. These have to be studied in the future. An example is the review on tissue-specific transcriptional profiles of NK cells and group 1 innate lymphoid cells, which are so far studied only in mice (Lopes et al. 2022).

Comparable to the ontogeny of the human spleen, there is also a sequence of development to splenic compartments in patients after bone marrow transplantation. In a group of 24 (median age 32 years) marrow recipients, the recovery of splenic white pulp compartments was studied (Nakayama et al. 1993). Up to 3 months, the white pulp was nearly atrophic, consisting mainly of T lymphocytes. The difference to ontogeny is that in ontogeny, the first phase is an accumulation of T lymphocytes in an undefined compartment.

### Isolated normothermic perfusion of the human spleen

#### Lymphocyte proliferation and cell cycle parameters in the human spleen

**Fig. 4:**
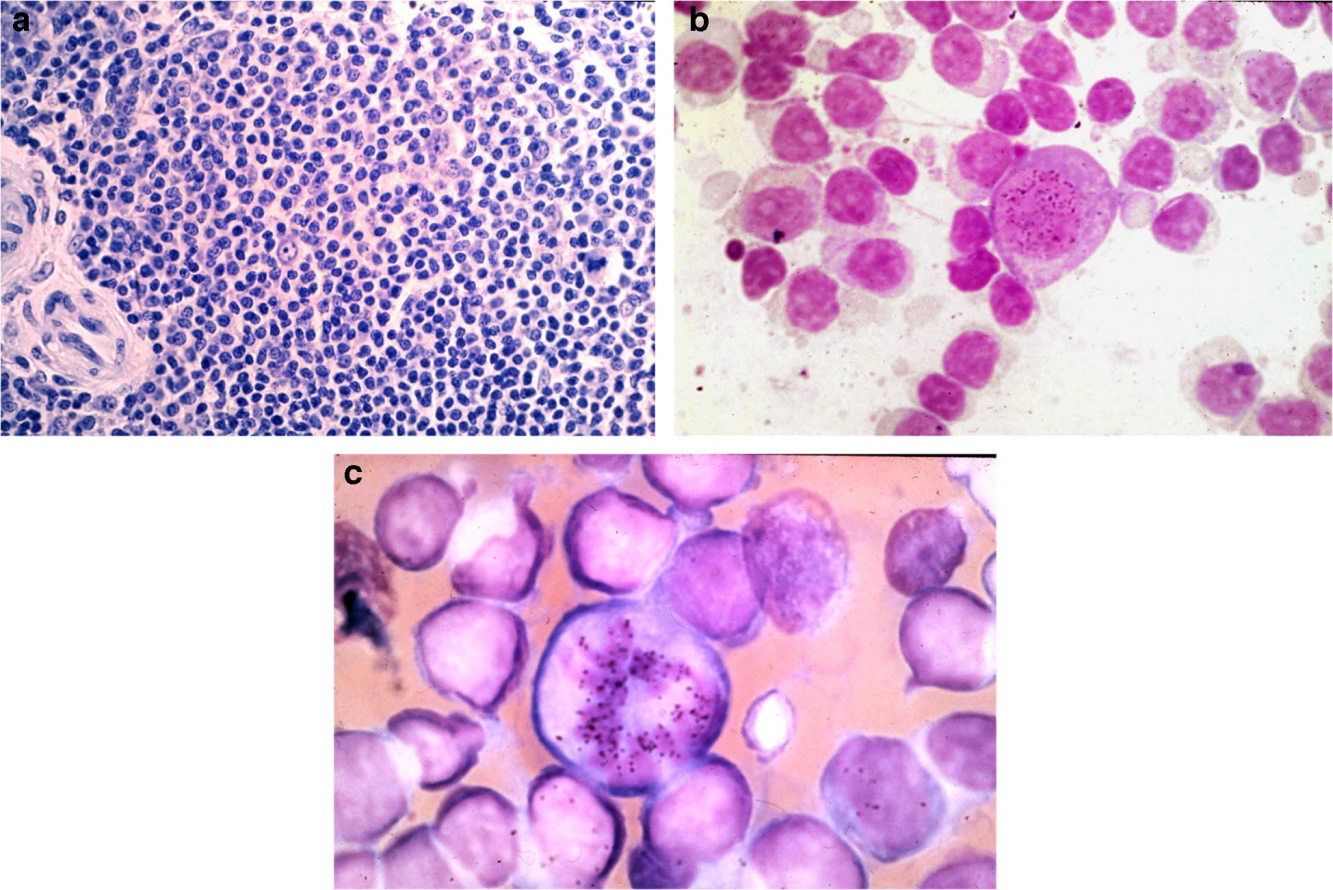
**a** Histology of a normothermic increase perfused human spleen 12 h after start. A central artery on the left and a mitotic figure on the right are obvious (magnification 400×). **b** A ^3^H-Tdr labeled immunoblast in the prophase of the cell cycle 1 h after flash labeling (magnification 2000 ×). **c** Labeled immunoblast labeled mitosis metaphase (magnification 2000 ×)

The technique of normothermic long-term perfusion of the isolated spleen was established in pigs, and the proliferation of lymphocytes was compared to the in vivo situation. This perfusion system was applied to human spleens obtained from patients with gastric cancer. The splenic structure was well preserved (Fig. 4A). The radioactive percussor of the DNA tritiated thymidine (^3^H-TdR) was added to the splenic artery either as a single pulse or continuously. Biopsies from these spleens were taken, and smears of these were evaluated by autoradiography. Examples of autoradiographs are shown in Fig. 4B and C. The mean labeling index for immunoblasts was 73% and for plasmoblasts 52%. The labeling index for small lymphocytes increased from 0 to 1% up to 28% at 14 h after ^3^H-TdR. The duration of the S-phase, determined by double labeling, was 8.3 h for lymphoid blast. Data from the labeled mitosis curve resulted in a minimal TG2 of about 1 h and tG2 and T14 of about 4 h. The results of hyperplastic spleens were similar to the normal spleen (Pabst and Reinecke 1981). The limitations of these experiments were that we cannot say where in the spleen their proliferation happens, as we used smears only.

## Factor VIII from the isolated perfused spleen

A further interesting finding of the normothermic isolated perfused human spleen was that in the perfusate factor VIII coagulant activity and factor VIII-related antigen increased. The total amount of factor VIII coagulant activity was equivalent to that of 3.5-l human plasma. It is not known whether factor VIII is stored as produced in the human spleen (Pabst et al. 1977).

## Splenectomy

**Fig. 5:**
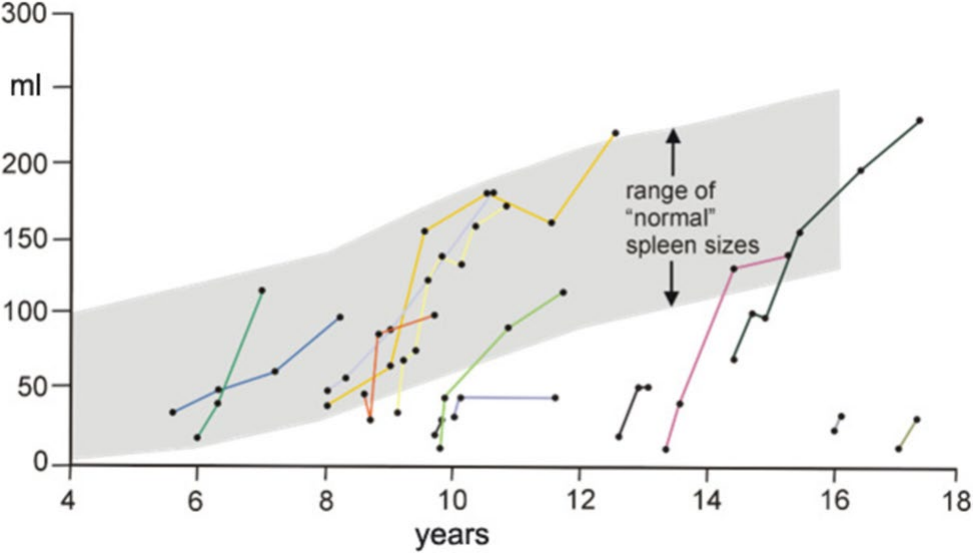
Growth of splenic remnants after partial splenectomy with spherocytosis in 14 patients (s. Eber Munich pers. Communication)

In hereditary spherocytosis, the spleen filters and destroys the malformed red cells, resulting in hemolysis, with resulting high bilirubin titers and gallstones. Therefore, splenectomy is indicated. A compromise is a near-total splenectomy, leaving some functional splenic tissue for immune reactions and reduction of destroying the pathologic red blood cells. Stoehr et al. (2006) documented the outcome of 31 patients with a moderate splenic regrowth dependent on disease state (Fig. 5). The same group (Stoehr et al. 2005) documented a sufficient antibody response to a pneumococcal vaccination and to meningococcal vaccination (Stoehr et al. 2008).

## Splenic autotransplantation

The clinical role of the spleen had been questionable for a very long time. Sherman (1980) discussed the perspectives in the management of trauma to the spleen in his presidential address of the American Association for Surgery of Trauma in 1980.

After splenic trauma, it has to be carefully documented how much of the spleen is ruptured to assess the amount of splenic tissue. The concepts to treat after splenic trauma have changed over the years. The age of the patient is critical because the incidence of OPSI is age dependent: 4% in children and 1% in adults (Reihneŕ and Brismar 1995). Surveillance imaging with CT is an important new technique: non-operative management has a success rate of 90%. However, depending on the grade of injury, splenic artery pseudoaneurysm has to be identified (Wallen et al. 2022). Splenectomy in hematological diseases is completely different. Partial splenectomy is the method of choice (Stoehr et al. 2008). The postoperative increase in the size of the splenic remnant is shown in Fig. 5**.**

After splenic rupture, e.g., after trauma, not only blood but also tiny fragments of splenic tissue can later be found at many sites, in particular in the abdominal cavity. This is called “splenosis peritonei.” These splenic nodules are sometimes found by chance, e.g., during an abdominal surgery at later times, e.g., a cholecystectomy. The spenules are rare but can be of clinical relevance as they can grow and restore some functions of the spleen. This is of interest to inform the patient. The presence and activity of this splenic tissue can be tested by the absence of Howell-Jolly bodies and pitted red cells (de Porto et al. 2010). There is some debate about how much splenic tissue is needed. In patients with more than 16% pitted red cells, the splenic volume will be equivalent to 22 to 133 cm^2^ splenic tissue (Corazza et al. 1984). The authors, therefore, recommend transplanting splenic tissue of 20–30 cm^2^. I would like to disagree because it is not the volume implanted but the functional splenic mass after regeneration in autotransplantation.

The regeneration of autotransplanted splenic tissue after a first phase of necrosis has been studied in detail in rats and pigs (Pabst et al. 1991). The advantage of implanting one’s own splenic tissue is that this is an autotransplantation and it will not be rejected. After a phase of necrosis, the red pulp regenerates first, followed by the white pulp and marginal zone. This is the sequence similar to ontogeny.

However, there are still many open questions in splenic autotransplantation: amount of transplanted tissue and time of function of the regenerated tissue. There is a communication of two cases of fatal pneumococcal septicemia despite ectopic splenic tissue 3 g and 92 g, respectively, of 8 and 9 years after splenectomy for trauma (Rice and James 1980).

In the clinics, spleen autotransplantation has been performed and the function assessed by CT and scintigraphy (Di Carlo et al. 2012) and different techniques (Badawy et al. 2022). Surendran et al. (2020) reviewed many studies. In previous studies, splenic function was restored, as documented by spleen scintigraphy, and in 90% of the cases of Ig, reached normal values. However, it has until now not been proven whether this regenerated splenic tissue is sufficient to prevent OPSI. Therefore, also in all cases of autotransplantation of splenic tissue, all prevention, like vaccination, has to be ordered by the doctors. Surveillance imaging with CT is an important new technique in non-operative management that has a success rate of 90%. However, dependent on the grade of injury, splenic artery pseudoaneurysms have to be identified (Wallen et al. 2022). Lesions of the spleen are often found incidentally by cross-section imaging, e.g., to exclude tumors. More complicated techniques are essential, such as computer tomography or magnetic resonance imaging. Krähling et al. (2024) have recently summarized such different lesions, as described in a flow chart for such diagnostic procedures.

Alternatives to splenectomy of the ruptured spleens are summarized in Table 1.

**Table 1:** Alternatives to splenectomy in the management of the ruptured spleen

Non-operation observation, e.g., ultrasound
Hemostatic agents, e.g., gelatine foam, thrombin
Arterial ligation, e.g., segmental artery, arterial embolism
Splenic rhaphy, e.g., mattress sutures, omental wrap absorbable not
Partial splenectomy, e.g., stapling, laser sutures
Partial angioembolism
Total splenectomy: autotransplantation of splenic tissue

### Side effects of splenectomy

#### Non-immune logical complication after splenectomy

Robinette and Fraumeni (1977) documented effects of splenectomy after trauma (*n* = 740) in the Second World War. Thrombocytosis and hypercoagulability may be the reason for the increased rate of fatal myocardial infection. The risk of cancer was not different. A very surprising and frequently reported vascular complication of splenectomy for hematological disorders is pulmonary hypertension [70]. Thromboembolic complications in splenectomy for thalassemia intermedia might be due to chronic intravascular hemolysis and ineffective erythropoiesis (Crary and Buchanan 2009). The morbidity and mortality following elective splenectomy for benign and malignant hematological disorders revealed that preoperative albumin levels and increased age seem to be relevant (Bagrodia et al. 2014). To prevent portal and caval thrombosis, prolonged anticoagulant therapy has been recommended (Buzelé et al. 2016).

#### Immunological side effects

The most important side effect of a splenectomy is sepsis. The surgical removal of the spleen can be essential, as in life-saving procedure after splenic trauma or indicated by a hematological disorder like spherocytosis and immune thrombocytopenia (for review, see Tahir et al. 2020). The incidence of splenectomy has been estimated to be about 6 per 100,000 people per year. The most relevant complication of splenectomy is the overwhelming postsplenectomy infection syndrome (OPSI) initially described by King and Shumacker in 1952. OPSI has a prevalence of 0.1–0.5% and a mortality of up to 50%. Encapsulated bacteria, after entering the blood, can only be removed by functioning splenic tissue. In man, immunoglobulin memory B cells essential to control *Streptococcus pneumoniae* infection are generated in the spleen (Kruetzmann et al. 2003). The most important bacteria are pneumococci, *Haemophilus influenzae* type B, and *Neisseria meningitidis*. Tahir et al. (2020) summarized the most important aspects to prevent death due to OPSI, such as patient education, vaccination, and antibiotic prophylaxis (Newland et al. 2005). A splenectomized patient should not travel to tropical areas and carry an identity card with the note “I am splenectomized” similar to patients with diabetes or those on anticoagulant drugs. All this seems to be of even greater relevance in infants because the marginal zone of the spleen, the most relevant compartment to filter bacteria, develops within the first 6 months of life (Kruschinski et al. 2004). Kibler et al. (2022) studied the age dependence of splenic structure in five organ donors, aged 3 to 48 years, in comparison to six splenic biopsies (= to 87 years of age). The human splenic marginal B cell pool changed with age, with a major population of lowly Ig-mutated CD27 negative but antigen-experienced B cells in early life (Kibler et al. 2021).

In splenectomized patients, the IgM and IgG antibody levels against pneumococcal antigens remained stable; however, the IgG specific memory cells were low. After vaccination, their Ig specific memory cell was similar to splenic children (Rosado et al. 2013). The splenectomized patients’ IgM memory B cells were decreased (Di Sabatino et al. 2004). These data clearly document the relevance of vaccination. Thirty-nine patients with spherocytosis were treated by near-total or total splenectomy and were sequentially vaccinated with a 23-valent pneumococcus polysaccharide vaccine, resulting in a relevant antibody response. It was documented that there was no difference between total and subtotal splenectomy (Stoehr et al. 2005). Rosado et al. (2013) documented in seven children that a postsplenectomy vaccination with antipneumoccic polysaccharide also resulted in a similar concentration of antibodies to a splenic child.

In conclusion, all patients with the indication of a splenectomy due to a hematological disorder should be vaccinated before the operative procedure, and splenectomized patients after splenic trauma have been vaccinated at least against pneumococci and carefully informed about the prevention of OPSI. Careful clinical observation of patients, e.g., by ultrasound in intensive care units, can avoid splenectomy in many cases or other surveillance imaging techniques (Wallen et al. 2022). The reader is recommended a review on the non-operative management of adults and children by worldwide experts in emergency surgery (Podda et al. 2022).

## Conclusions and future dissection

The human spleen is a clinically relevant organ and should be preserved as much as possible. Total splenectomy can often be prevented by modern surgical techniques, e.g., partial splenectomy. Whenever a spleen has to be removed, the patient has to be vaccinated, e.g., against pneumococci to prevent an overwhelming postsplenectomy infection (OPSI) which is often lethal. Future techniques might be available to test the function of the red pulp and white pulp, replacing the traditional determination of Howell-Jolly bodies or pitted red cells. New markers will characterize NK cells and, to prevent OPSI, innate lymphocytes in the human spleen. It has to be studied whether anticoagulant therapy is indicated after splenectomy.

## Data Availability

No datasets were generated or analysed during the current study.
